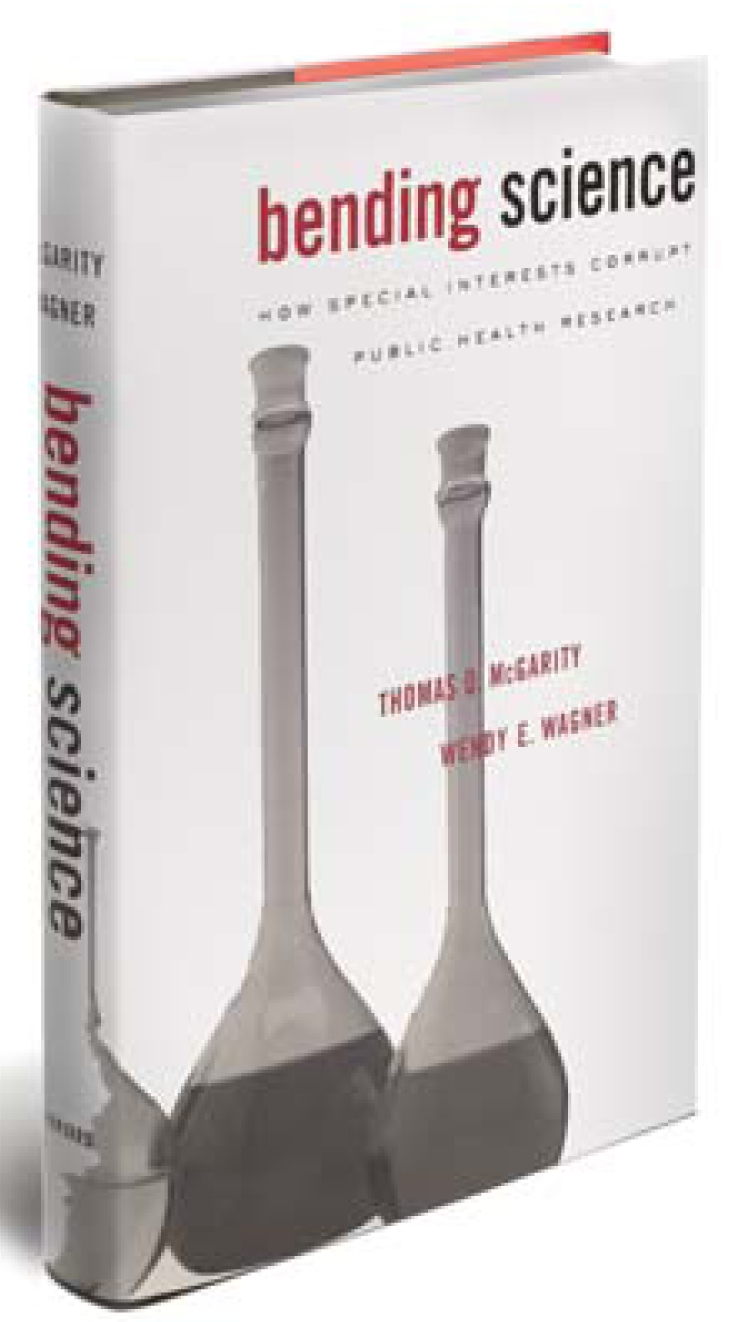# Bending Science: How Special Interests Corrupt Public Health Research

**Published:** 2008-11

**Authors:** Jennifer Sass

**Affiliations:** Jennifer Sass is a scientist at the Natural Resources Defense Council (NRDC), working to strengthen federal oversight of hazardous chemicals, pesticides, and nanomaterials. Sass directs the scientific integrity project at NRDC. She has published more than two dozen articles, served on federal advisory committees, and provided testimony to the U.S. Congress and National Academies

Biased reporting of science has been documented for industry-supported research on many hazardous substances, including the plasticizer bisphenol A, secondhand tobacco smoke, asbestos, and lead. Several books that have hit the stands recently (e.g., David Michaels’ *Doubt Is Their Product*) use case studies to document and discuss the effect this kind of bias has on public health and environmental protection.

In *Bending Science* McGarity and Wagner discuss the methods and motivations that make this practice so pervasive. The book could be called “Idiot’s Guide to Bending Science” because its chapters neatly and logically provide a step-by-step plan for manipulating science to support a predetermined conclusion. Starting with who has an interest in the manipulation of science, the book describes how to distort science without getting caught, how to support “bent” science by attacking legitimate science and scientists, and finally how to use public relations firms and journalists to advertise and disseminate the “bent” science. In addition to “how,” the book tells us why manufacturers and other financially interested parties are motivated to manipulate science—namely, to weaken the regulation of their products and to defend themselves in litigation if harm comes from their products.

A recent illustration of the impact of “bent science” on public health is evident in the Food and Drug Administration’s (FDA) draft assessment of bisphenol A issued this summer, declaring the chemical was safe as currently used. The FDA’s assessment relied on just two studies, which were funded by the American Chemistry Council (formerly the Chemical Manufacturers Association), Dow Chemical, Bayer, and other plastics manufacturers, and the agency ignored dozens of other studies done by independent scientists that reported evidence of harm. The FDA’s conclusions also conflict with two National Institutes of Health reviews and the actions of its counterpart in Canada.

An example of the failure of our regulatory oversight mechanisms to provide a backstop was evident this summer when Congress was compelled to pass legislation to eliminate lead in children’s toys and to ban or temporarily suspend the use of six types of phthalates (components of plastics) in children’s products. Congress stepped in after regulatory agencies failed to take action, even though children had been widely exposed (one child died in March 2006 from lead-contaminated toys) and there was substantial scientific evidence that these chemicals were highly hazardous.

*Bending Science* has a halting academic writing style that overly relies on secondary sources as resources. In addition, the authors argue that everyone bends science, even public health advocates; however, the few public health examples that the authors provide are relatively rare instances that do not support those sweeping conclusions. For example, a case study of plaintiffs’ lawyers artificially inflating silicosis cases fails to mention that this was a highly unusual instance for which the offending lawyers were issued sanctions for their transgressions. In fact, without trial lawyers much of the evidence that the authors rely on for this book, such as the tobacco industry documents, would have never been released for public scrutiny.

This is a topic of great importance. *Bending Science* warns that when science becomes artificially manipulated to misrepresent the hazards of products, “serious adverse consequences for human health and the environment, as well as for the economic well-being of legitimate businesses,” may arise.

## Figures and Tables

**Figure:**